# A Facile Method for Preparation of Cu_2_O-TiO_2_ NTA Heterojunction with Visible-Photocatalytic Activity

**DOI:** 10.1186/s11671-018-2637-8

**Published:** 2018-07-24

**Authors:** Yulong Liao, Peng Deng, Xiaoyi Wang, Dainan Zhang, Faming Li, Qinghui Yang, Huaiwu Zhang, Zhiyong Zhong

**Affiliations:** 1 State Key Laboratory of Electronic Thin Film and Integrated Devices, University of Electronic Science and Technology of China, Chengdu, 610054 China; 2 Center for Applied Chemistry, University of Electronic Science and Technology of China, Chengdu, 611731 China

**Keywords:** TiO_2_ nanotube, Cu_2_O, Heterojunction, Thermal decomposition, Visible photocatalysis

## Abstract

Based on highly ordered TiO_2_ nanotube arrays (NTAs), we successfully fabricated the Cu_2_O-TiO_2_ NTA heterojunction by a simple thermal decomposition process for the first time. The anodic TiO_2_ NTAs were functioned as both “nano-container” and “nano-reactors” to load and synthesize the narrow band Cu_2_O nanoparticles. The loaded Cu_2_O expanded absorption spectrum of the TiO_2_ NTAs from ultraviolent range to visible light range. We found that the Cu_2_O-TiO_2_ NTA heterojunction films had visible activity towards photocatalytic degrading methyl orange (MO). The photocatalytic abilities of the Cu_2_O-TiO_2_ NTA heterojunction films were found increased with the Cu_2_O content from 0.05 to 0.3 mol/L. This could be explained by more electron-hole pairs generated and less recombination, when the Cu_2_O-TiO_2_ heterojunction got formed. Here, we put forward this promising method, hoping it can facilitate the mass production and applications of Cu_2_O-TiO_2_ NTA heterojunction.

**Electronic supplementary material:**

The online version of this article (10.1186/s11671-018-2637-8) contains supplementary material, which is available to authorized users.

## Background

With more and more attention paid to the environmental issues nowadays, the study of water treatment materials emerged in a continuous stream [1–4]. Hundreds of strategies were proposed for the treatment of polluted water. However, there were many problems, such as low efficiency, low recycling rate, and secondary environment pollution, restricting their further applications [5–7]. The semiconductor materials were considered to be a promising candidate, and titanium oxide was recognized as one of the best photocatalyst materials due to its high photocatalytic activity and good chemical and mechanical stability [8–12]. Recently, TiO_2_ materials with nanotube (NT) array were widely studied, and the tubular morphology was proved to be a promising structure for photocatalysis. Compared with other microcosmic morphologies, TiO_2_ NT arrays owned several significant advantages [13–17]. Firstly, unique tubular structure could enhance electron-transporting efficiency and restrain the recombination of carriers, which will further produce more reactive oxygen species (ROS) [18, 19]. Secondly, TiO_2_ NT arrays are much easier to recycle than the TiO_2_ powder photocatalysts [20–24]. Thirdly, TiO_2_ NT arrays have large specific surface area and high surface energy. However, due to the relatively wide gaps (~ 3.2 eV). TiO_2_ NT photocatalyst is only active under UV irradiation [25–28]. In fact, a photocatalyst that is able to respond with visible light will surely take obvious advantages. At present, the focus of photocatalyst research is to adjust their light response band and improve their photocatalytic efficiency.

**Fig. 1:**
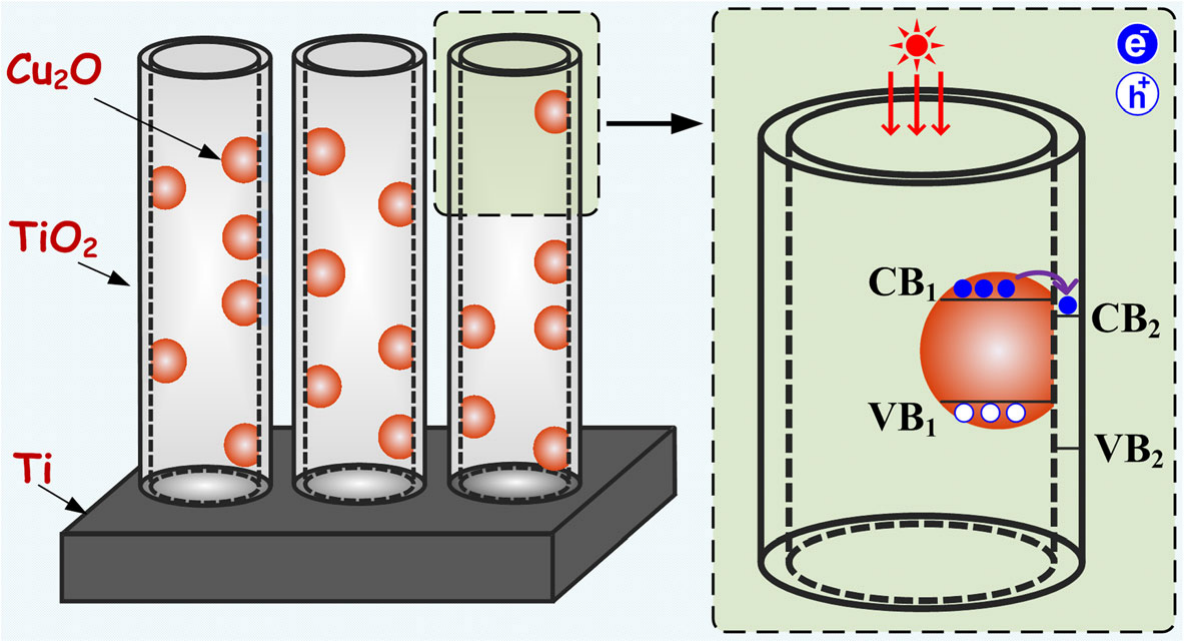
Schematic structural diagram of Cu_2_O-TiO_2_ NTA heterojunction. Under visible light illumination, the electrons were excited to the conduction band of Cu_2_O particles and then transferred to the conduction band of TiO_2_ for the matching band gap structure

Building heterogeneous TiO_2_ photocatalysts with narrow band gaps is one of the hotspots as an attempt to overcome such impediments. Narrow band semiconductors, like Cu_2_O, CdS, CdTe, PbS, and Bi_2_O_3_, have been studied to build TiO_2_ heterojunction photocatalysts [29–34]. Among them, Cu_2_O (with the direct gap of ~ 2.2 eV) is regarded as one of the best candidates. For Cu_2_O, the response band is about 560 nm, and its band gap structure happens to well match with the energy level of TiO_2_ NTs. As schematically shown in Fig. 1, under the excitation of visible light, electron/hole pairs are generated and the photoinduced electrons are excited to the conduction band of Cu_2_O and then transfer to the conduction band of TiO_2_, which suppresses the recombination of electrons and holes. This heterojunction structure solves the problem that the TiO_2_ materials could not respond to visible light and the problem that electron/hole pairs generated on Cu_2_O get recombined easily. From this point of view, Cu_2_O-TiO_2_ NTA heterojunction structure materials guaranteed a natural advantage in visible light photocatalysis.

General approach to prepare Cu_2_O-TiO_2_ heterojunction photocatalysts includes chemical coprecipitation and electrodeposition, and the products have shown promising photocatalytic performances. But it is still a challenge to prepare Cu_2_O-TiO_2_ heterojunction photocatalysts with good quality by using a facile and low-cost method. Inspired by the concept of the precursor from Chemical vapor deposition (CVD), the idea, using acetate to carry copper ion to get into the inside of TiO_2_ NTs prepared by anodic oxidation, comes out. It is known that metal organic compounds are likely to get thermally decomposed. In this study, anodic TiO_2_ NTAs were functioned as “nano-container” to load copper acetate at first and then as “nano-reactors” to provide space for thermal decomposing the loaded copper acetate. After a thermal treatment, Cu_2_O-TiO_2_ TNA heterojunction films were successfully obtained. To the best our knowledge, this method has not been reported to prepare Cu_2_O-TiO_2_ TNA heterojunction. Furthermore, the phase composition, morphology, and photocatalytic activity were characterized by XRD, EDS, SEM, and spectrophotometer.

## Experimental Section

The chemicals which were mentioned in the experiment process were purchased (Sinopharm Group Chemical Reagent Co. Ltd., China) and used without further purification, except the deionized water with a resistance of 18.3 MΩ cm.

### Preparation of Pure TiO_2_ Nanotube Arrays

Anodic oxidation method was used to prepare uniform and stable TiO_2_ NTAs with vertical alignment [35, 36]. Metal titanium (Ti) sheets were cut into pieces of 1.5 × 5 cm^2^ and cleaned by a cleanser. After a sonication bath in ethanol, the Ti pieces were dried in oven. Electrolyte consisted of 535.45 g glycol, 10 g deionized water, and 1.6617 g NH_4_F, which were mixed and stirred for 2 h. Then, we took two pieces of Ti as anode and cathode, respectively. Immersing them into electrolyte, applying a constant potential of 50 V for 2 h, amorphous TiO_2_ nanotube arrays (TiO_2_ NTAs) were fabricated at room temperature.

### Synthesis of Cu_2_O-TiO_2_ NTA Heterojunction

**Fig. 2:**
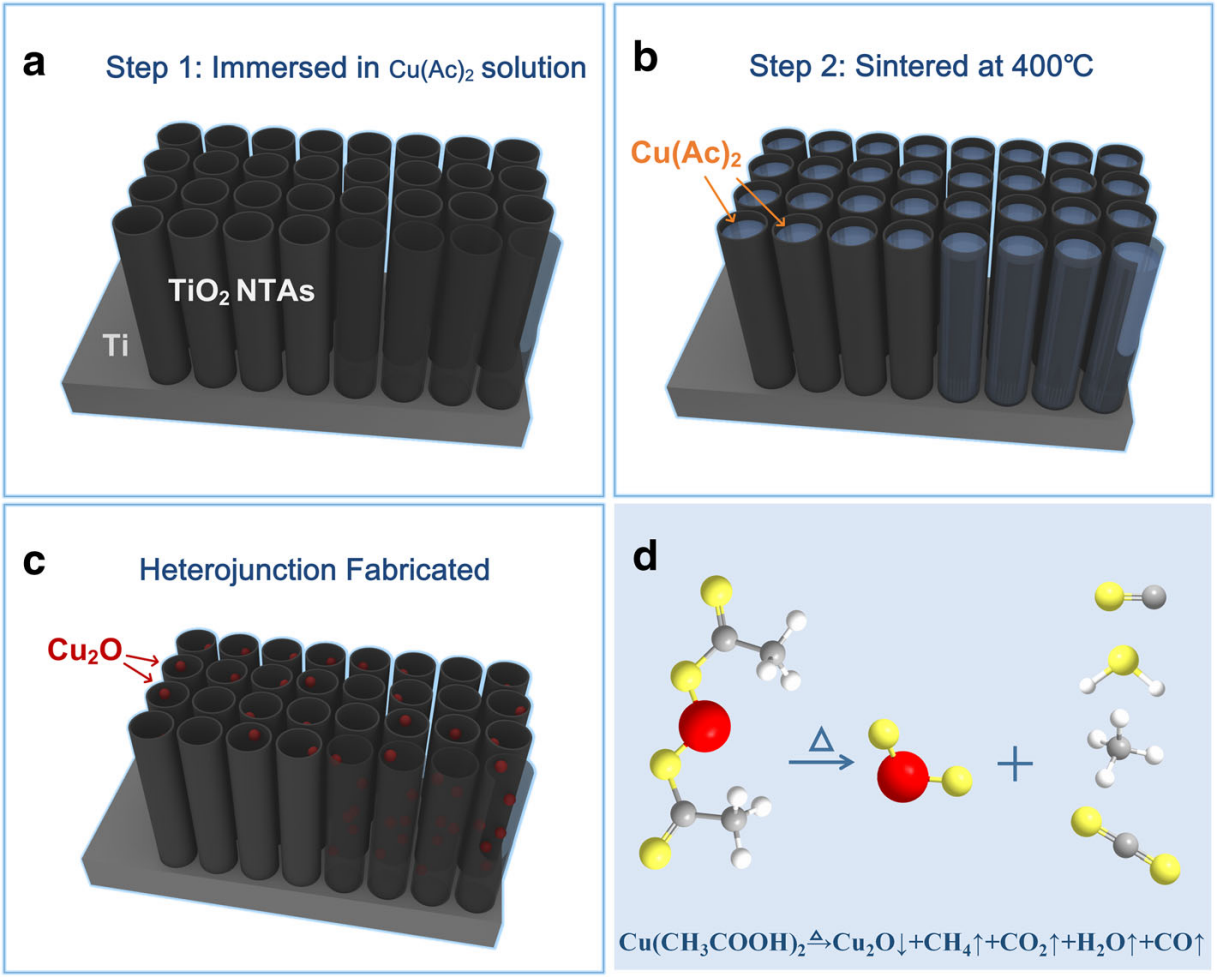
Synthesis procedure of the Cu_2_O-TiO_2_ NTA heterojunction films. **a** Step 1, anodic TiO_2_ NTAs. **b** Step 2, fill the tubes with precursor solution. **c** Step 3, sintered the filled tubes at 400 °C to get the Cu_2_O-TiO_2_ NTA heterojunction. **d** Chemical reaction formula of the sintering process

The amorphous TiO_2_ NTAs were crystallized into anatase by a thermal treatment at 450 °C. And then, they were used as substrate to prepare Cu_2_O-TiO_2_ NTA heterojunction film. First, cupric acetate (Cu(Ac)_2_) with different concentration was prepared, ranging from 0.05 to 0.3 mol/L. Then, annealed TiO_2_ NTAs were immersed into the solution transiently and dried in oven at 70 °C immediately. And the final products, Cu_2_O-TiO_2_ films, were marked as sample S1-S5 respectively by the different Cu(Ac)_2_ concentration of 0.05, 0.1, 0.2, 0.3, and 4 mol/L in this immersing process. After this process, the cupric acetate molecules had got into the TiO_2_ nanotubes. Next step was putting the samples into an atmosphere-sintering furnace of N_2_ with a sintering temperature of 400 °C for 150 min. The cupric acetate was thermal decomposed in a way described by Eq. (1). Finally, the Cu_2_O-TiO_2_ NTA heterojunction films were prepared. This process was schematically shown in Fig. 2.

1 $$ {\left({\mathrm{CH}}_3\mathrm{COO}\right)}_2\mathrm{Cu}\overset{\Delta}{\to }{\mathrm{Cu}}_2\mathrm{O}\downarrow +{\mathrm{CH}}_4\uparrow +{\mathrm{CO}}_2\uparrow +{\mathrm{H}}_2\mathrm{O}\uparrow +\mathrm{CO}\uparrow $$

Just like holding a test tube containing cupric acetate, after heating, cupric acetate thermal decomposed into Cu_2_O which was left inside the TiO_2_ NTAs.

### Characterization

A scanning electron microscopy (SEM, JSM-7000F, JEOL Inc., Japan) with energy dispersive spectrometer (EDS) was used for the observation of the morphology and structure. The samples were characterized by a D/max-2400 X-ray diffraction spectrometer (Rigaku, D/max-2400, Japan) and a UV-vis spectrometry (Ultrospec 2100 pro) was also used. To evaluate the photocatalytic activity of the as-synthesized Cu_2_O-TiO_2_ NTA heterojunction, we took methyl orange (MO), a typical organic indicator, as the degraded object. The Cu_2_O-TiO_2_ NTA films (3.0 × 1.5 cm^2^) were immersed in 5 × 10^−5^ mol/L of MO aqueous solution and irradiated with seven 4 W visible bulbs (Toshiba, Cool white, FL4W, Japan). Then, the solution was magnetically stirred in the dark for 30 min to ensure adsorption-desorption equilibrium prior to photocatalytic degradation. Photodegradation experiments lasted 180 min with 1.5 mL samples withdrawn periodically. The concentration of the residual MO was measured by a spectrophotometer at about 460 nm on the basis of the Beer-Lambert law. The degradation efficiency of the MO could be defined as follows:

2 $$ {C}_t/{C}_0=\left({A}_t/{A}_0\right)\times 100\% $$

And the varying of *A*_*t*_/*A*_*0*_ referred to the changing in *C*_*t*_, which represented the photocatalytic activity of the tested samples.

## Results and Discussion

**Fig. 3:**
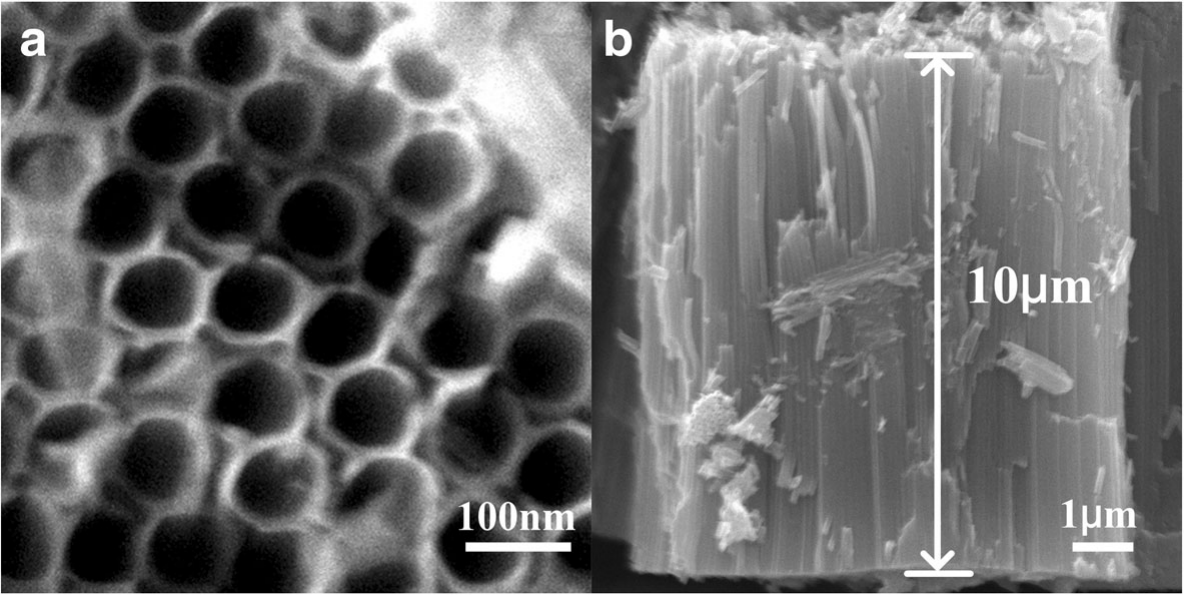
Typical SEM images of pure TiO_2_ nanotube arrays without modification. **a** Top view and **b** side view, indicating the highly ordered vertical alignment structure with open-tube mouth morphology. The tube diameter is about 100 nm, and the tube length is about 10 μm

**Fig. 4:**
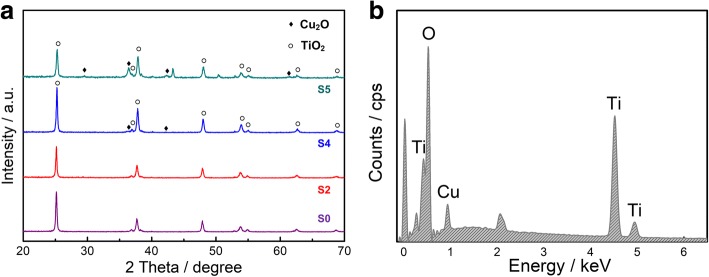
**a** XRD patterns of the Cu_2_O-TiO_2_ NTA heterojunction samples. Sample S0: pure anatase TiO_2_ NTA sample; samples S2, S4, and S5: thermally decomposed sample with immersing in 0.1, 0.3, and 4 mol/L Cu(Ac)_2_ solution, respectively. **b** EDS result of the Cu_2_O-TiO_2_ NTA heterojunction films, showing the existence of elements Ti, Cu, and O. The results confirm the successful loading of Cu_2_O on the TiO_2_ NTAs

Figure 3 shows a typical SEM observation of the pure anodic TiO_2_ NTAs after annealing at 450 °C. Anodizing is an electrolytic process which converts the outer surface of metals into an oxide layer or pore structure. As shown in Fig. 3, the as-prepared TiO_2_ NTs have open-tube morphology with a uniform outer diameter distribution of ∼ 100 nm. The anodic TiO_2_ NTAs are highly ordered and oriented, and each single TiO_2_ NT owns very smooth tube walls with an average thickness of ∼ 10 nm. Our former studies have shown that tube length, the diameter, and morphology could be manipulated by adjusting the anodization protocols [37, 38]. The SEM results also indicate the thermal annealing at a high temperature of 450 °C does not destroy morphologies of the TiO_2_ NTAs. XRD is used to characterize the crystalline of the pure TiO_2_ NTAs (sample 1), see Fig. 4a. Results show that diffraction peaks locating at 25.3°, 36.9°, 37.8°, 48°, 53.9°, 55°, 62.7°, and 68.8° could be observed in sample 1, attributing to the (101), (103), (004), (200), (105), (211), (204), and (116) of anatase phase, respectively. As we know, there are three types of titanium dioxide phase, anatase, brookite, and rutile. Rutile could show relatively good photocatalytic ability with a granularity less than 10 nm. However, to get a rutile phase, the TiO_2_ sample needs to be heated up to a high sinter temperature of 800 °C, which could lead to the break of TiO_2_ tubes in this case. Brookite phase is hardly to be formed by using thermal annealing method for the bad thermodynamic phase stability, while anatase is the most common phase with good photocatalytic activity [39, 40]. The sharp diffraction peaks and the strong intensity of sample 1 (see Fig. 4a) indicated a highly crystallized anatase structure, which meant our TiO_2_ substrate was excellent not only in the morphology but also in the crystalline phase. The highly ordered TiO_2_ NTAs with open-tube mouth morphology were used as substrate to prepare Cu_2_O-TiO_2_ NTA heterojunction films in this study.

XRD patterns of the TiO_2_ NTAs loaded with Cu_2_O nanoparticles in concentration gradient ranging from 0.05 to 4.0 mol/L are also shown in Fig. 4a, and the 4.0 mol/L sample was prepared by a cycling immerse process described in Additional file 1, the “Experimental Details” part. The samples were named samples 2 to 4 with the increasing Cu(Ac)_2_ concentration. Except the TiO_2_ peaks, there was no peak of Cu_2_O showing up in sample 2 because of the tiny amount of the loading Cu_2_O particles. And particles might be decorated inside of the TiO_2_ “nano-container” which also raised the difficulty for characterization. In sample 3 and sample 4, obvious cuprite peaks could be observed at 29.6°, 36.4°, 42.3°, and 61.3°, attributing to the cuprite (110), (111), (200), and (220) of Cu_2_O, respectively. It should be noted here that sample 4 was only used to characterize the existence of Cu_2_O particles, and its synthetic details were described in Additional file 1. Moreover, the lattice parameters and the grain size were calculated based on the XRD data. After removing the background and K_α2_ diffraction, and following the smoothing and fitting process, we got the average lattice parameters of our samples of *a* = *b* = *c* = 4.2646 Å, which matched with the standard PDF. The standard PDF showed that the lattice parameters of Cu_2_O are: *a* = *b* = *c* = 4.2696 Å, and Cu_2_O had a cubic structure [41]. The average grain size of Cu_2_O was calculated as ~ 47 nm, by using Debye-Scherrer formula:

3 $$ D=\frac{K\gamma}{B\cdot \cos \theta } $$

In Eq. (3), *D* is the grain size, *K* is the Scherrer constant, *γ* is the wavelength of X-ray, *B* is FWHM which needs to be in the radian, and *θ* is the diffraction angle. XRD results indicate that the Cu(Ac)_2_ were loaded into the TiO_2_ NTAs and successfully decomposed into Cu_2_O inside the same TiO_2_ NTAs, and then the Cu_2_O-TiO_2_ NTA heterojunction films got formed. To further investigate the Cu_2_O-TiO_2_ NTA heterojunction, an elemental analysis was carried out by using EDS. Figure 4b showed an EDS diagram of Cu_2_O-TiO_2_ NTA heterojunction film which was prepared with 0.2 mol/L Cu(Ac)_2_. The atomic percentages were 7.32, 28.96, 57.45, and 6.27% for elements Cu, Ti, O, and impurity C. This result showed that the Cu_2_O owned a relatively low content in the heterojunction sample, but it still brought about the visible-light activity, which would be discussed later in the MO degradation experiment. The EDS results well agreed with the XRD results in Fig. 4a that cuprite Cu_2_O was successfully loaded to anatase NTAs.

**Fig. 5:**
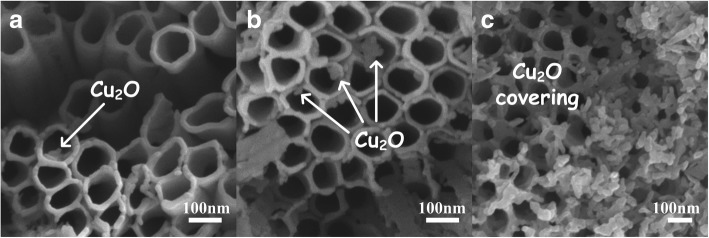
Typical SEM images of the Cu_2_O-TiO_2_ NTA heterojunction films. **a** Sample immersed in 0.2 mol/L Cu(Ac)_2_. **b** Sample immersed in 0.3 mol/L Cu(Ac)_2_. **c** Sample immersed in Cu(Ac)_2_ of over high concentration

Figure 5 showed the top view SEM results of the modified TiO_2_ NTAs. Compared with the pure TiO_2_ NTA samples in Fig. 3, a few small particles could be seen near the upper and inside of the TiO_2_ tubes in Fig. 5a. Increasing the modification amount, a number of nanoparticles could be observed obviously in Fig. 5b. Figure 5c was sample 4 that we discussed before. Large parts of the tube surface were covered by the redundant Cu_2_O, indicating that sample 4 was over-decorated. Based on the SEM images, the size distribution of Cu_2_O particles was estimated ranging from ~ 30 to ~ 80 nm, which well agreed with XRD calculated grain size of ~ 47 nm. For the tubular structure of the three samples, they still retained the vertical alignment state, but some tubes got a little awry. It was considered as the influence of thermal decomposition process, which needed a heating process of 400 °C to get Cu(Ac)_2_ decomposed into Cu_2_O. High temperature in the decomposition step had a negative effect on the tubular structure, supported by the SEM images. However, if the heating temperature in the thermal process went too low to 240 °C, Cu(CH_3_COO)_2_·H_2_O would just get dehydrated instead of decomposed. So the temperature should be controlled in ~ 300 to 400 °C to keep the nano-scale tubular structure and ensure the fabrication of Cu_2_O-TiO_2_ NTA heterojunction. It can be concluded that the Cu_2_O-TiO_2_ heterojunction could be formed, and the morphology retained well, when the decomposition happens at 400 °C.

**Fig. 6:**
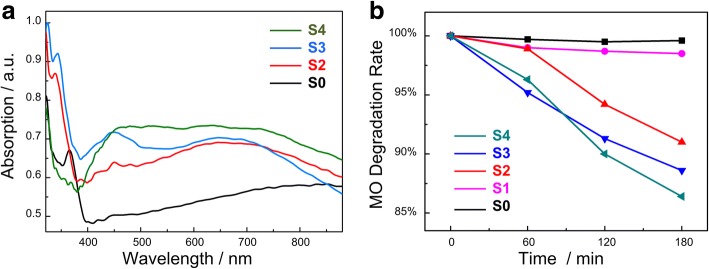
**a** UV-vis spectra of the Cu_2_O-TiO_2_ NTAs and absorption get expanded to visible light range and raised with the loading amount of Cu_2_O. **b** Visible-light photocatalytic degradation kinetics of MO treated by the heterojunction films with different Cu_2_O content. When decoration magnitude of Cu_2_O on TiO_2_ NTAs increased, the MO decomposition efficiency under visible light irradiation raised. Sample S0 referred to pure TiO_2_ film, and sample S1–S5 were the samples immersed in Cu(Ac)_2_ solution with the concentrations of 0.05, 0.1, 0.2, 0.3, and 4 mol/L, respectively

The Cu_2_O nanoparticles were loaded on TiO_2_ NTAs to fabricate the heterojunction, which was expected to enhance the photo-response ability in visible light range, so UV-vis characterization was adopted to investigate optical properties of the as-synthesized Cu_2_O-TiO_2_ NTAs. Figure 6a shows the UV-vis absorption spectra of the Cu_2_O-TiO_2_ NTA samples with Cu_2_O-loaded magnitude increasing from none to 4.0 mol/L. It could be seen in Fig. 6a that the pure TiO_2_ NTAs without loading Cu_2_O only showed out a high absorption in the ultraviolet region (< 380 nm), due to its intrinsic material properties. After loading the Cu_2_O particles, the absorption range was expanded to 600–700 nm. And when the intensity is increasing with the raising of the Cu_2_O modification magnitude, the absorption value of the Cu_2_O-TiO_2_ heterojunction films also got increased. Figure 6a indicated that TiO_2_ NTAs were given the visible light response ability by decorating Cu_2_O nanoparticles. UV-vis along with SEM, EDS, and XRD results proved that the Cu_2_O-TiO_2_ NTA heterojunction was fabricated successfully by the thermal decomposition method, and samples showed the enhanced visible light absorption.

**Fig. 7:**
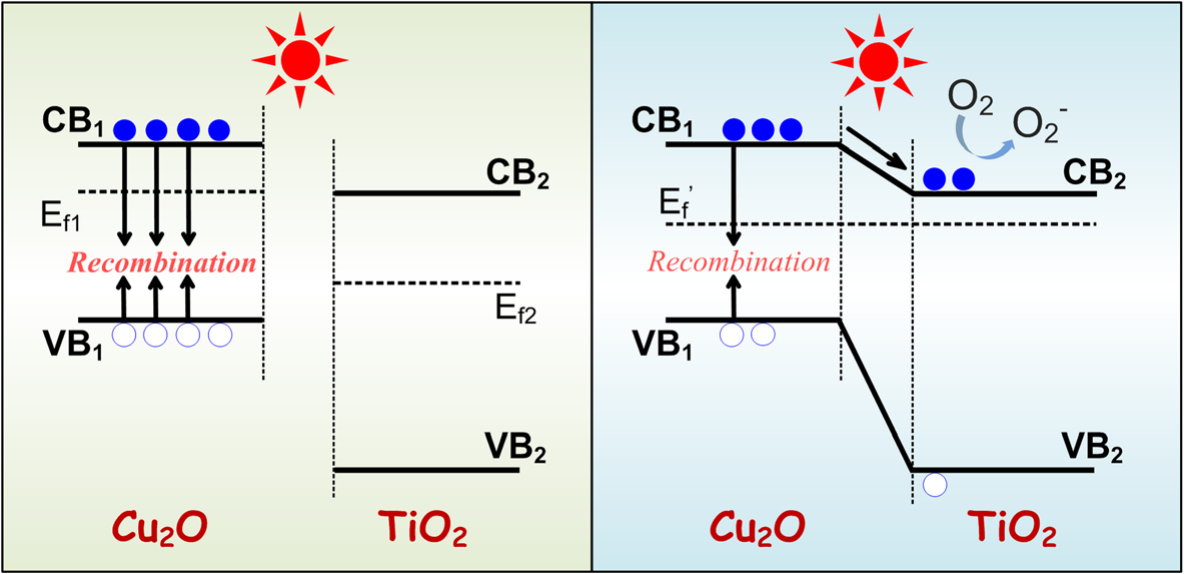
Band gap structure of Cu_2_O and TiO_2_ before (left) and after (right) contact. When the Cu_2_O-TiO_2_ heterojunction is formed, the electron/hole pairs photogenerated on Cu_2_O could transfer to TiO_2_ NTAs

Photocatalytic activities, one of the most important properties of the Cu_2_O-TiO_2_ NTA films, were evaluated through degradation of MO aqueous solution. The visible-light photocatalytic degradation kinetics was shown in Fig. 6b. The MO degradation rate was proportion to the loading amount of Cu_2_O approximately. The more Cu_2_O particles were loaded on TiO_2_ NTAs, the faster MO got degraded. Sample S1 degraded MO to 91.0% in 3 h under visible light irradiation, while sample S4 degraded MO to 86.4% in 3 h under the same condition. MO degradation rate represented the photocatalytic activity of the samples. Comparing with the photocatalytic degradation rate to the ~ 2.73% of CdTe-TiO_2_ by a pulse electrodeposited method [29], ~ 45% of Bi_2_O_3_ by a ultrasonication-assisted successive ionic layer adsorption and reaction (SILAR) technique [32], and ~ 27.25% of Cu_2_O by a square wave voltammetry method [33], photoactivity of this as-synthesized Cu_2_O-TiO_2_ sample was improvable. However, as a facile new strategy, it still conduced to improve fabrication method. When the Cu_2_O loading amount went up, there was a trend that the photocatalytic activity of our as-synthesized Cu_2_O-TiO_2_ NTA heterojunction films increased. It indicated the Cu_2_O content had a positive influence of the visible-light photocatalytic activity. TiO_2_ itself only responded to ultraviolet, and the visible light range photocatalytic ability should come from the decoration of Cu_2_O. As shown in Fig. 7, the conduction band bottom of Cu_2_O was a little higher than that of TiO_2_, while the valence band top of Cu_2_O was higher than that of TiO_2_. So, the photoinduced electrons were excited to the conduction band of Cu_2_O and then transferred to the conduction band of TiO_2_. As a direct-gap semiconductor, wave vector of Cu_2_O was just the same at the bottom of the conduction band and the top of valence band. It meant that only the changes of energy were required, instead of the changes of momentum. This energy band structure led to the situation that carriers recombined easily. However, due to the help of heterojunction structure, the photogenerated electrons on Cu_2_O transferred to TiO_2_ NTAs which suppressed the recombination of electron/hole pairs. The longer the pairs existed, the more easily the ROS got produced which brought this photocatalytic activity. As more Cu_2_O loaded on TiO_2_ NTAs, the heterojunction fabricated better. And the photocatalytic ability got promoted. So, the Cu_2_O content showed a positive influence of the visible-light photocatalytic activity. However, further increase of Cu_2_O content as well as the photocatalytic ability is limited, due to the solubility of Cu(Ac)_2_ in aqueous solution which was 7.2 g (0.36 mol/L) at room temperature. And sample S5 with Cu(Ac)_2_ concentration of 4.0 mol/L is prepared by a cycling immerse process described in Additional file 1, the Experimental Details part. The photocatalytic degradation of the MO followed pseudo-first-order kinetics [42] and the kinetic reaction could be expressed as:

4 $$ {A}_t={A}_0{e}^{- kt} $$

While our degradation curve showed nearly a straight line, it is not an exponential function. So, there was still room for improvement. And the solubility limit could be broken by the repeated immersion method we mentioned before, with further investigation of the Cu(Ac)_2_ concentration and repetition times to avoid adverse effects. In this study, as this thermal decomposition method was what we concerned and tried to illustrate, we just took the 0.3 mol/L (close the solubility of 0.36 mol/L) as the maximum concentration of Cu(Ac)_2_ solution. And the photocatalytic activity in visible light range of our as-synthesized heterojunction was confirmed by the MO degradation results. Our previous study found that the Degussa P25 had similar ultraviolet photocatalytic activities with TiO_2_ NTAs, when the power P25 was placed on a glass substrate [28]. It can be concluded that we have successfully prepared Cu_2_O-TiO_2_ NTA heterojunction films with visible-light photocatalytic activities.

## Conclusions

In summary, we have successfully prepared the Cu_2_O-TiO_2_ NTA heterojunction films by a simple thermal decomposition process. SEM, EDS, and XRD results show that TiO_2_ NTAs with a tube diameter of ~ 100 nm were loaded by Cu_2_O nanoparticles with an average size of ~ 50 nm. The anodic TiO_2_ NTAs functioned as both “nano-container” and “nano-reactors” to load and synthesize the narrow-band Cu_2_O nanoparticles, which has not reported before. UV-vis spectra indicate that the absorption range of the TiO_2_ NTAs was expanded from ultraviolent range to visible light range, due to the loading of Cu_2_O. Photocatalytic testing indicated that there was a visible-light photocatalytic activity of the as-synthesized Cu_2_O-TiO_2_ heterojunction. The photocatalytic abilities of the Cu_2_O-TiO_2_ NTA heterojunction films were found to be increased with the Cu_2_O content from 0.05 to 0.3 mol/L. Our current work has demonstrated a novel and facile method to prepare Cu_2_O-TiO_2_ NTA heterojunction films, which could also be promising for environmental and energy-related areas.

## Additional file

Additional file 1: The experimental details of preparing the Cu_2_O-TiO_2_ samples and further characterization results of Raman spectra and XRD patterns are provided as the supplemental information to support the discussion. (DOCX 2387 kb)

## Abbreviations

EDS: Energy-dispersive spectrometry
NTAs: Nanotube arrays
SEM: Scanning electron microscopy
XRD: X-ray diffraction
