# Supplementary material for: A Facile Method for Preparation of Cu2O-TiO2 NTA Heterojunction with Visible-Photocatalytic Activity
Source: Nanoscale Res Lett. 2018 Jul 24;13:221. doi: 10.1186/s11671-018-2637-8 (PMC6057862; doi:10.1186/s11671-018-2637-8)
Supplement: Supplementary file 1 — The experimental details of preparing the Cu2O-TiO2 samples and further characterization results of Raman spectra and XRD patterns are provided as the supplemental information to support the discussion. (DOCX 2387 kb) [file 11671_2018_2637_MOESM1_ESM.docx]

**Additional file 1**

A Facile Method for Preparation of Cu_2_O-TiO_2_ NTAs Heterojunction with Visible-Photocatalytic Activity

Yulong Liao,^[[1]](#footnote-1)^*^[a],[b]^ Peng Deng,^[a]^ Xiaoyi Wang,^[a]^ Dainan Zhang,^[a]^ Faming Li,^[b]^ Qinghui Yang,^[a]^ Huaiwu Zhang,^[a]^ and Zhiyong Zhong^[a]^

*^[a]^* State Key Laboratory of Electronic Thin Film and Integrated Devices, University of Electronic Science and Technology of China, Chengdu 610054, China

*^[b]^* Center for Applied Chemistry, University of Electronic Science and Technology of China, Chengdu 610054, China

***Experimental Details:***

***Preparation:*** Samples were sintered twice. The first-step sintering transited the amorphous phase into anatase phase. The second-step sintering thermally degraded the Cu(Ac)_2_ into Cu_2_O and fabricated the heterojunction. The appearance of the samples after first-step sintering and the second-step sintering were shown in Fig. S1. The main processes were depicted as follows:

**(1)** First step was the preparation of pure TiO_2_ nanotube arrays. We cut the titanium foil in the exact 1.5×5 cm^2^ Ti pieces, and cleaned them with cleaner and sonication bath in ethanol to remove the grease stains and other stains. With a quick dry process at 60 ^o^C, the ethanol was evaporated. Then we prepared the electrolyte of 535.45 g glycol, 10 g deionized water and 1.6617 g NH_4_F. The pure TiO_2_ NTAs samples were fabricated by a typical electrochemical anodic oxidation. Anode and cathode were both Ti pieces with precise controlling of voltage, current density, pieces spacing, temperature and reaction time. All the reagents were analytical purity and were purchased from Sinopharm Group Chemical Reagent Co. Ltd. and Shanghai Aladdin Bio-Chem Technology Co., LTD, China. A DC power supply (Model GPS-3303c, GW Instrument Co., Ltd Taiwan) was employed to provide a constant potential of 50 V. Then highly ordered TiO_2_ nanotube arrays on the Ti sheet substrate could be obtained.

**(2)** Now we had the “tubes” to contain the precursor solution of Cu(Ac)_2_. Taking out the prepared Cu(Ac)_2_ aqueous solution of different concentration gradients (ranging from 0.05 mol/L to 0.3 mol/L), we immersed the as-obtained TiO_2_ films into the precursor solution and carefully filtered out excess solution with filter paper to avoid the to-be-produced Cu_2_O particles blocked the tube mouths. In this stage, the filter paper should not contact the films surface or just filtered the excess solution along the films edges. Then we obtained the samples of tubes filled with Cu(Ac)_2_ as shown in Figure 3(b). As there was a solubility limitation for Cu(Ac)_2_, 0.36 mol/L, it was difficult to increase the modification content of Cu_2_O. So if we wanted to get highly modified Cu_2_O-TiO_2_ NTAs samples, we would repeat the immerse step. It meant we immersed the pure TiO_2_ NTAs sample into Cu(Ac)_2_ solution and dried it normally, but we kept re-immersing it and drying it, instead of sintering directly. After re-immersing for 10 times, we finally put it into sintering furnace.

**(3)** Sintering Cu(Ac)_2_ into Cu_2_O and ensuring the fabrication of heterojunction were the most important and difficult part of our experiment. As mentioned before, this thermal degradation preparation method was inspired by the idea of using acid radical to carry the metal cation. After the acid radical thermal degraded, the metallic oxide or inorganic salt were left. But the thermal decomposition of organic compounds is very complicated and the decomposition products were always hybrid. So, we repeated experiments times to find the exact reaction parameters for our synthesis of heterojunction. For the bulk materials, the thermal degradation formulas were:

(CH_3_COO)_2_Cu·H_2_O → (CH_3_COO)_2_Cu 0 ^o^C ~150 ^o^C

(CH_3_COO)_2_Cu → Cu_2_O 150 ^o^C ~300 ^o^C

Cu_2_O → Cu 400 ^o^C ~450 ^o^C

And if samples were sintered in the air atmosphere, oxygen would oxidize them into copper(II) oxide. So this formula only worked in oxygen-free atmosphere. But in our experiment, we found that samples sintered at 350 ^o^C in nitrogen atmosphere were still copper(II) oxide rather than copper(I) oxide. And samples sintered at 400 ^o^C were with the phase of cuprite. However, in this batch of samples, we found there were metal copper existed in one of our heterojunction films (five samples in total). As the copper only appeared in one sample and the copper actually played a positive role in the photocatalytic activity, we decided to adopt this solution. And the final sinter parameters were 400 ^o^C sintering with a heating rate of 2 ^o^C/min in nitrogen atmosphere and heat preservation for 150min. After Cu_2_O-TiO_2_ NTAs heterojunction synthesized, polycrystalline Cu_2_O was difficult to be oxidized. And the cuprite phase could keep for weeks.

***
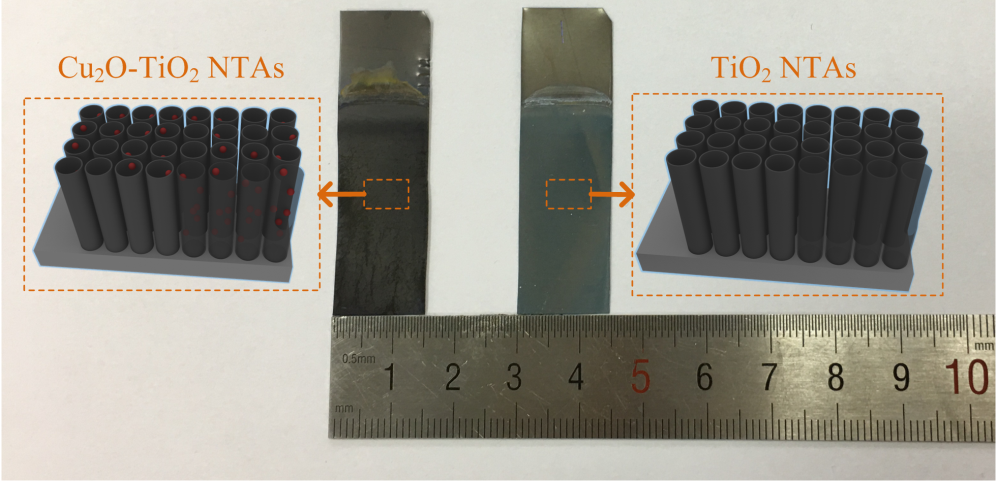
***

**Figure S1.** The appearance of samples. Right: The TiO_2_ NTAs without any modifying; Left: The Cu_2_O-TiO_2_ NTAs heterojunction.

***Characterization:*** EDS results confirmed the existence of element copper. XRD showed the phase structure of cuprite. SEM (JSM-7000F, JEOL Inc. Japan) indicated the combination of TiO_2_ nanotube arrays and Cu_2_O particles. Spectrophotometer data of MO degradation revealed the photocatalytic activity of our heterojunction samples. The phase structure of the as-sintered samples were examined by an X-ray diffractometer (XRD: Rigaku, D/max-2400, Japan), operated at 40kV and 30mA, at a scanning rate of 3.6 ^o^/min and a step of 0.03 ^o^ in the range of 20^o^≤2θ≤70^o^, using CuKα radiation.

***Photocatalytic testing:*** The photocatalytic activities of the as-synthesized Cu_2_O-TiO_2_ NTAs heterojunction films evaluated on the basis of the degradation of methyl orange (MO, 5×10^-5^ mol/L) as model organic pollutants in aqueous solutions. Samples (1.5×3 cm^2^ surface area, total mass ~0.04 g) were immersed in the solution and irradiated with seven 4 W visible bulbs (Toshiba, Cool white, FL4W, Japan). Then the solution was magnetically stirred in the dark for 30 min to ensure adsorption-desorption equilibrium prior to photocatalytic degradation. Photodegradation experiments lasted 180 min with 1.5 mL samples removed periodically. The concentration of the residual MO was measured by a spectrophotometer at about 460 nm on the basis of the Beer-Lambert Law. It was important to note that visible bulbs could radiate out the ultraviolet. A filter glass is needed to block the ultraviolet light in order to monitor the visible light photocatalytic activity of our as-synthesized heterojunction samples.


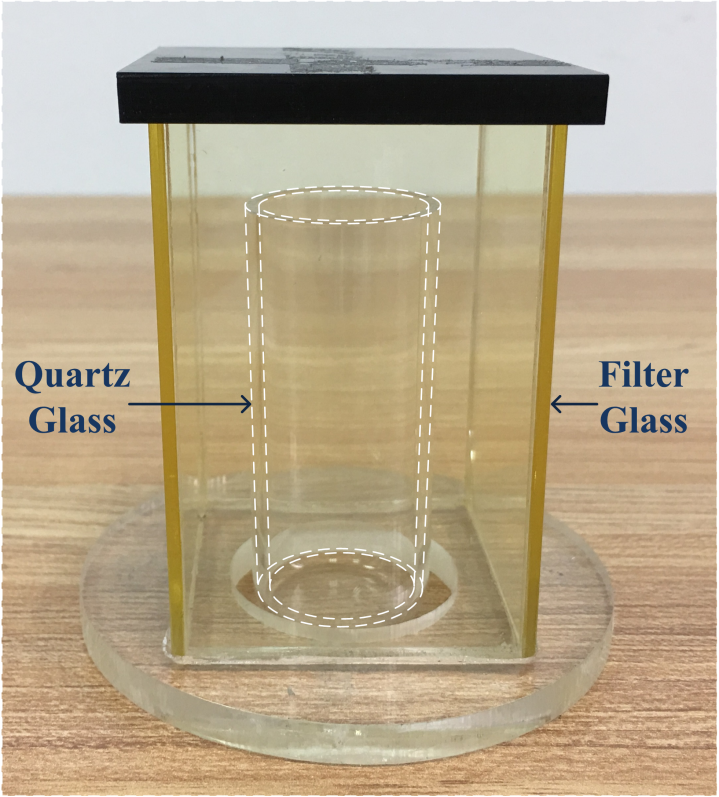


**Figure S2.** Photocatalytic experimental unit. The quartz glass would be filled with MO solution and MO would get degraded by the photocatalytic ability of Cu_2_O-TiO_2_ heterojunction. When the light was illuminating, filter glass would remove the ultraviolet to avoid the influence from high-energy ultraviolet.


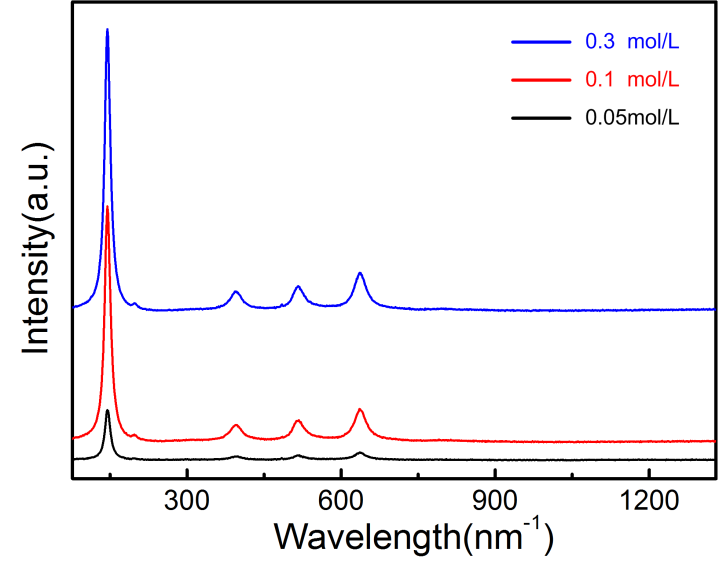


**Figure S3.** Raman spectra (inVia, Renishaw, UK) results. None of them showed the Cu_2_O peaks. All the peaks (at 141 nm^-1^, 396 nm^-1^, 516 nm^-1^ and 633 nm^-1^) belonged to TiO_2_. We considered it as a result of Cu_2_O existing in tubes mainly, instead of the surface. Cu_2_O Raman peaks should exist at 224 nm^-1^, 419 nm^-1^, 518 nm^-1^ and 635 nm^-1^ which were almost covered by TiO_2_ peaks. And there was just a feeble modification of Cu_2_O rather than heavy doping. So it was hard to detect the small peaks of Cu_2_O.


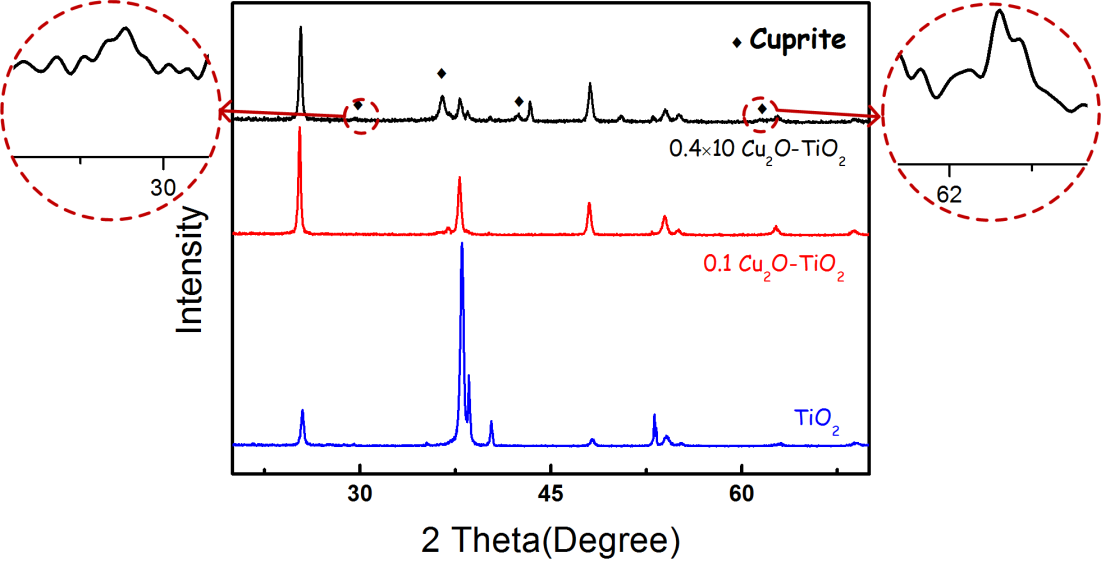


**Figure S4.** Typical XRD results of unmodified TiO_2_ NTAs sample, Cu_2_O-TiO_2_ NTAs heterojunction sample that was immersed in Cu(Ac)_2_ solution of 0.1mol/L before the second-step sinterin, and the Cu_2_O-TiO_2_ NTAs heterojunction sample had the re-immersing steps before second-step sintering. The 0.4×10 Cu_2_O-TiO_2_ sample was used to characterize the existence of element Cu and the phase Cuprite, and it wouldn’t be used in the photocatalytic activity experiment process.

1. * Corresponding author:

   Tel.: +86-028-83201440; Fax: +86-028-83202556

   Email address: yulong.liao@uestc.edu.cn [↑](#footnote-ref-1)
